# *Dysaster cajamarcensis*, a new shrubby genus and species of Astereae (Asteraceae) from Peru

**DOI:** 10.3897/phytokeys.36.7393

**Published:** 2014-04-25

**Authors:** Harold Robinson, Vicki Funk

**Affiliations:** 1 Department of Botany, MRC 166, NMNH, P.O. Box 37012, Smithsonian, Washington, DC. 20013-7012

**Keywords:** Asteraceae, Astereae, *Diplostephium*, Hinterhuberinae, Peru

## Abstract

*Dysaster cajamarcensis* is a spreading broad-leaved shrub named as a new genus and species of the tribe Astereae subtribe Hinterhuberinae collected in northern Peru. It has bisexual disc florets, disc style branches with strong stigmatic lines and hairy appendages, compressed achenes in both ray and disc florets, and papyraceous involucral bracts.

## Introduction

There is something very unsatisfying about a plant, sent for identification, that has no strikingly distinctive feature, but has a combination of characteristics that excludes it from any already known genus. It is particularly unsatisfying when the plant involved is a member of a tribe such as the Astereae in which phyletic studies using DNA (Brouillet et al. 2009) are not yet adequately correlated with morphological and anatomical studies. Nevertheless, such a plant has been collected in northern Peru. The specimen of the broad-leaved shrubby plant arrived with a field identification of *Diplostephium*, the latter a genus of shrubby Astereae that is common in the Central and Northern Andes. The plant is not a *Diplostephium* Kunth, and has characteristics that do not agree with any other genus in the tribe.

Attempts to identify the plant have involved the use of keys in Hoffmann (1890–1894), Cuatrecasas (1969), Nesom and Robinson (2007), Strother and Brouillet (2006), and lists of genera and species in Braco and Zarucchi (1993), and the genera sequenced in the treatment by Brouillet et al. (2009). Results were as follows.

The treatment by Brouillet et al. (2009) includes no unaccounted for elements among the listed South American Astereae. More importantly, all of the Astereae listed in the Catalogue of the Flowering Plants and Gymnosperms of Peru (Brako and Zarucchi 1993) and in the treatment of the tribe in Colombia (Cuatrecasas 1969) can be excluded. The keys to genera in various treatments are not much more helpful. In Hoffmann (1890–1894) the plant keys into the relationship of *Sommerfeltia* Less., but the latter is a distinctive element from southeastern South America with deeply dissected leaves. In Cuatrecasas (1969) the new entity keys to *Aster* L., a concept that in that work was based on two introduced species now known to be *Symphyotrichum* Nees. The Peruvian plant also keys to the *Symphyotrichum* relationship in Nesom and Robinson (2007), but the involucral bracts are totally non-herbaceous. When keyed among North American genera in Strother and Brouillet (2006), the Peruvian plant comes to *Ampelaster* G.L. Nesom, another member of the *Symphyotrichum* relationship.

One further possibility exists. The involucral bracts have a median dark stripe that might be indicative of the resin duct characteristic of the subtribe Conyzinae. Among the genera of that subtribe, the new entity would key in Nesom and Robinson (2007) to *Darwinothamnus* Harling. The latter is endemic to the Galapagos Islands, and it is a linear-leaved rather ericoid-looking plant with inflorescences not or scarcely exserted. It has chaffier, more recurved involucral bracts and small narrow limbs on the ray florets. The achenes in *Darwinothamnus* are sparsely setuliferous on the faces rather than densely spiculiferous, and the marginal ribs contain enlarged resin ducts. The pappus lacks a well-defined outer series, and bristles have tenuous rather than broadened tips.

A comparison on a broad scale using preliminary DNA sequencing (ITS1 & 2) places the new entity among previously sequenced Astereae that are almost all members of the subtribe Hinterhuberinae. The genera that show closest correlation are *Hinterhubera* Sch. Bip. ex Wedd., *Parastrephia* Nutt., *Guynesomia* Bonifacino & Sancho, the diminutive epappose *Laestadia* Kunth ex Less., and *Diplostephium*. Of these, *Hinterhubera* is an ericoid, mostly narrow-leaved genus of Colombia and Venezuela that has narrow corolla lobes and functionally male disc florets. *Parastrephia* is a genus of cupressiform resinous shrubs with bisexual disc florets and nearly terete achenes from mostly southern Peru, Bolivia and Chile. *Guynesomia* is a plant with sparse linear leaves, bisexual florets and scarcely compressed achenes that is endemic to Chile. There remains *Diplostephium* which is the only genus in the group that has species that are remotely similar in habit to the unknown entity from northern Peru. None of these show DNA correlation closer than 97%.

In spite of all the results from various keys and DNA results, it is the genus *Diplostephium* in which the Peruvian plant was placed by the collectors, and it is that genus with which it is most likely to be confused on brief observation. The new genus and *Diplostephium* differ in five significant characteristics.

(1) The achenes of the new entity are compressed with only two ribs in both ray and disc florets;

(2) The disc florets are fully bisexual with style branches having well-developed stigmatic lines;

(3) Involucral bracts are narrowly lanceolate and sharply pointed with a dark median stripe outside;

(4) the outer pappus is a strongly differentiated series of squamae; and

(5) the inflorescence is exserted well beyond the foliate parts of the branches and has few heads on long peduncles.

*Diplostephium* has more triangular and prismatic achenes, functionally male disc florets lacking stigmatic lines on their style branches, involucral bracts that are more ovate, less pointed, and without an external median stripe, a less strongly differentiated outer pappus series that has shortened bristles of variable lengths, and an inflorescence that is usually dense and mostly sessile, rarely subumbellate.

The Peruvian entity is named here as new at both the generic and species level.

## Taxonomic treatment

### Dysaster cajamarcensis

H. Rob. & V.A. Funk gen et sp. nov.

urn:lsid:ipni.org:names:77138096-1

http://species-id.net/wiki/Dysaster_cajamarcensis

#### Type.

Peru. Dept. Cajamarca: Prov. Contumazá. 14 km S of Contumazá on gravel road, rocky slopes, Western Cordilleran evergreen forest, 2520 m, 17 Jul 1992, *T.F. Stuessy, D.W. Crawford & A. Sagástegui 12686* (holotype US; isotypes OH, HUT).

Shrubs with spreading branches, with scattered upright branchlets ca. 2 dm long; stem surfaces densely white tomentose, internodes mostly 5–10 mm long. Leaves alternate, sometimes with small axillary fascicles. Petioles ca. 5 mm long; blades elliptical, 1.0–2.5 cm long, 0.4–0.8 cm wide, bases cuneate, margins with 5–8 small teeth, apices obtuse to subacute, upper surface dark green with some arachnoid tomentum, bullate with veins insulcate, lower surface densely whitish tomentose with strongly exsulcate veins; venation pinnate, with 5–6 veins on each side, spreading at ca. 45° angles. Inflorescences strongly excerted on tips of foliose branchlets, branching with usually 3 capitula; peduncles slender, 4–7 cm long, thinly whitish tomentose, with few scattered minute bracteoles above base. Capitula radiate, heterogamous, with rays to 3–4 cm wide; involucres campanulate, ca. 0.8 cm high, ca. 1.5 cm wide, bracts ca. 70 in 3–4 series, 2–8 mm long, ca. 0.8 mm wide, linear-lanceolate with slender tips, narrowly scarious and often reddish at margins and tips, mostly papyraceous, pale greenish outside with dark longitudinal median stripe; receptacle epaleaceous. Ray florets 30–35, fertile, female; corollas pink-purple, basal tubes ca. 2.5 mm long, limbs ca. 12 mm long, 2 mm wide, scarcely trilobed at tip, without evident glands or trichomes except abaxially at base of limb, style branches with stigmatic lines continuous along margins and apex; disc florets ca. 75; corollas yellow, narrowly funnelform, ca. 7 mm long, basal tube ca. 2 mm long, glabrous, throat ca. 4.7 mm long, with some short, pointed, septate hairs near base, lobes ca. 0.7 mm long, oblong-ovate, with few short, septate hairs at tip; anther thecae ca. 2 mm long, slightly pointed at base with few sterile cells at base; anther appendage ca. 0.2 mm long; style base slightly broadened. Ray and disc achenes alike, ca. 2 mm long, lenticular, compressed with 2 costae along margins, costae not containing enlarged resin ducts, lateral surfaces densely covered with short spicules, setulae numerous near base, few setulae distally; pappus of ca. 17 slender bristles ca. 5 mm long, slightly broadened distally. Outer series of numerous scale-like squamae ca. 0.3 mm long. Pollen in fluid ca. 30 μm in diam.

#### Distribution.

Known only from the type from Cajamarca, Peru.

#### Ecology.

Rocky slopes, Western Cordilleran evergreen forest, elevation 2620 m.

#### Etymology.

*Dys-* – bad, + *aster* – for the genus.

**Figure 1.**
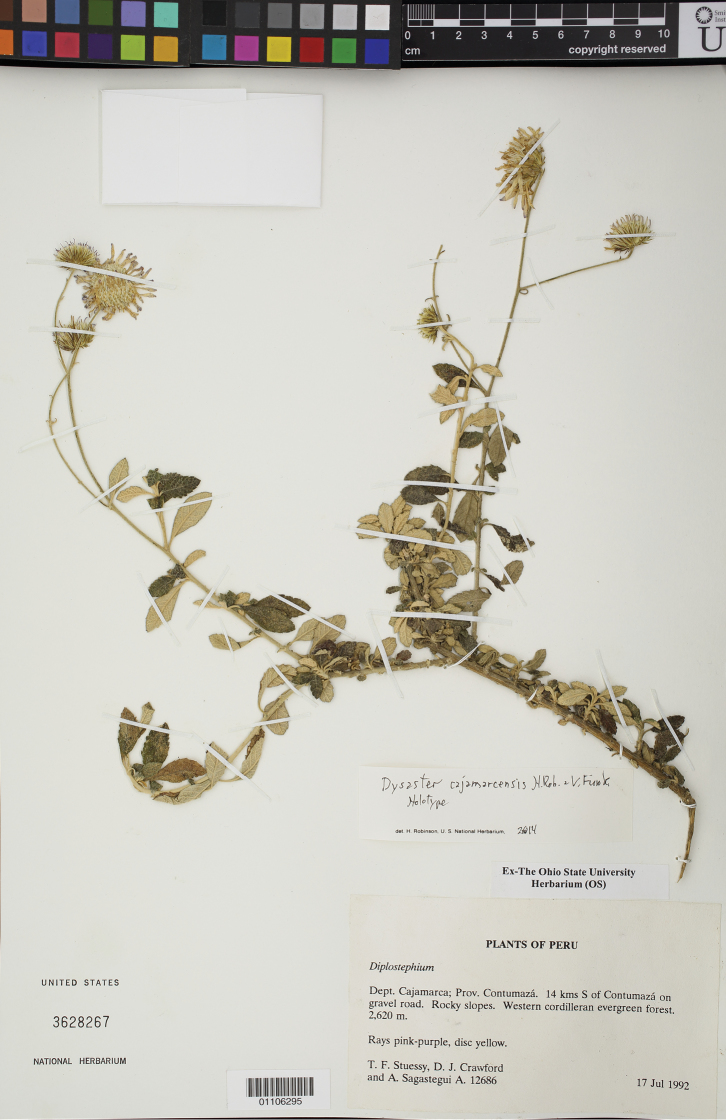
Holotype of *Dysaster cajamarcensis* H. Rob. & V.A. Funk (*Stuessy, Crawford & Sagastequi 12686*, US).

**Figure 2.**
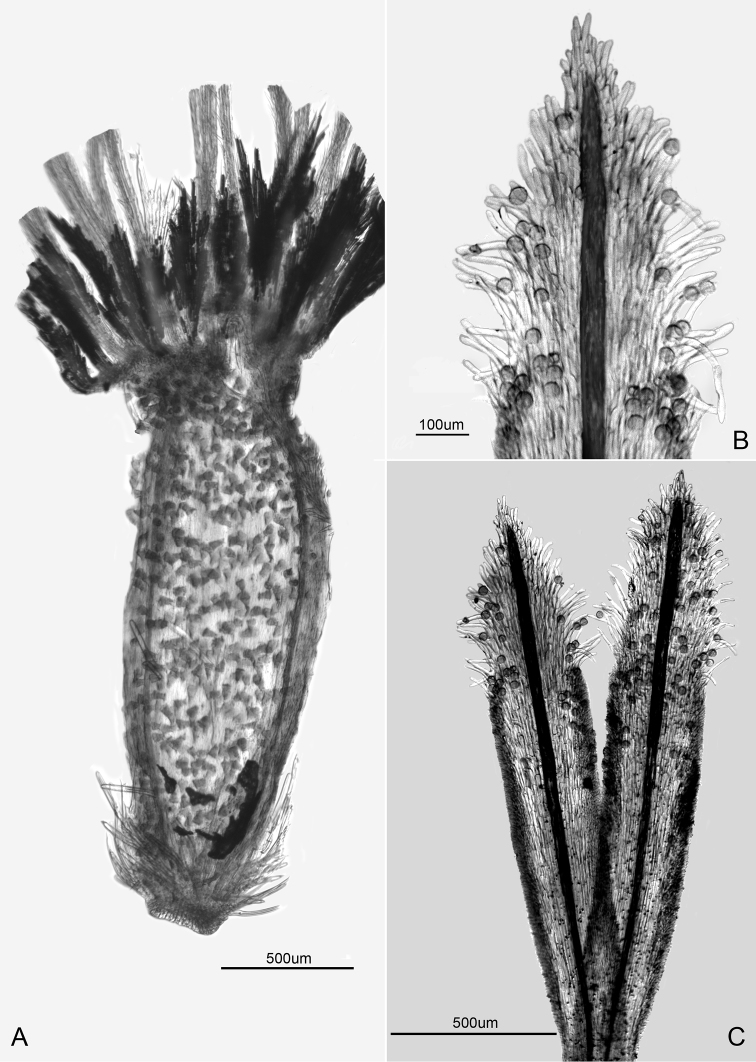
Floral details of *Dysaster cajamarcensis* H. Rob. & V.A. Funk **A** Disc achene **B** Style appendage **C** style branches showing stigmatic lines and appendages.

## Supplementary Material

XML Treatment for Dysaster cajamarcensis
